# The hidden burden of viral hepatitis B and C in healthcare settings: a cross-sectional study of seroprevalence, predictors, and occult infection

**DOI:** 10.3389/fpubh.2026.1964413

**Published:** 2026-10-15

**Authors:** Mikhail Kostinov, Olga Isaeva, Ivan Samolygo, Svetlana Erdes, Marina Manina, Albina Pestova, Anton Antishin, Polina Kondrashova, Asadi Mobarhan Fedor, Alexander Novikov, Elena Tanaschuk, Tatiana Androsova, Izabella Khrapunova, Andrey Linok, Marina Loktionova, Naida Magomedova, Nikolay Briko, Aristitsa Kostinova

**Affiliations:** 1 Department of Epidemiology and Modern Vaccination Technologies, Institute of Professional Education, I. M. Sechenov First Moscow State Medical University (Sechenov University), Moscow, Russia; 2 Laboratory of Vaccine Prevention and Immunotherapy of Allergic Diseases, Federal State Budgetary Scientific Institution “I. Mechnikov Research Institute of Vaccines and Sera”, Moscow, Russia; 3 Laboratory of Molecular Epidemiology of Viral Hepatitis, Central Research Institute of Epidemiology, Moscow, Russia; 4 Laboratory of Viral Hepatitis, Federal State Budgetary Scientific Institution “I. Mechnikov Research Institute of Vaccines and Sera”, Moscow, Russia; 5 Department of Propaedeutics of Children’s Diseases, N. F. Filatov Clinical Institute of Children’s Health, I. M. Sechenov First Moscow State Medical University (Sechenov University), Moscow, Russia; 6 Department of Internal, Occupational Diseases, and Rheumatology, N. V. Sklifosovsky Institute of Clinical Medicine, I. M. Sechenov First Moscow State Medical University (Sechenov University), Moscow, Russia; 7 Department of Social Hygiene, Organization of Surveillance, with a Course of Laboratory Diagnostics, Dagestan State Medical University, Makhachkala, Russia; 8 Department of Epidemiology and Evidence-Based Medicine, F. F. Erisman Institute of Public Health, I. M. Sechenov First Moscow State Medical University (Sechenov University), Moscow, Russia

**Keywords:** epidemiology, healthcare workers, hepatitis C, occult hepatitis B, occupational risk, PCR diagnostics, seroprotection

## Abstract

**Background:**

Bloodborne viral hepatitis B and C remain critical occupational hazards for healthcare workers (HCWs). Current mandatory screening in some countries, including Russia, relies solely on hepatitis B surface antigen (HBsAg) detection, which may overlook occult infections. This study aimed to evaluate the prevalence, predictors, and occupational risks of HBV (including occult HBV infection) and HCV among HCWs in multidisciplinary facilities.

**Methods:**

A cross-sectional observational study of 1,643 HCWs and auxiliary staff was conducted. Blood serum samples were tested for anti-HBc, anti-HBs, and anti-HCV antibodies using ELISA. Seropositive individuals underwent real-time PCR testing to detect HBV DNA and HCV RNA. Multivariable logistic regression was used to identify independent predictors of seropositivity.

**Results:**

Anti-HBc IgG was detected in 36.6% of HCWs, demonstrating a strong age gradient (aOR = 1.55 per 10 years of age; *p* < 0.001), rising from 12.4% in those under 30 to 50% in those over 60. Anti-HBs levels ≥10 mIU/mL were observed in only 54.8% of HCWs, dropping to 29.7% at a stricter threshold (>100 mIU/mL). High anti-HBs titers (100–1,000 mIU/mL) were strongly associated with anti-HBc positivity (aOR = 4.65; *p* < 0.001). Occult hepatitis B infection (OBI) was detected in 3.1% (95% CI 0.85–7.73%) of tested anti-HBc-positive HCWs, who exhibited lower median anti-HBs concentrations compared to PCR-negative counterparts (7 vs. 165 mIU/mL; *p* = 0.003). HBV DNA was detected in 4 of 129 HBsAg-negative, anti-HBc-positive participants (3.1%), a finding consistent with possible occult HBV infection. Notably, 75% of OBI cases were classified as low occupational risk. Anti-HCV prevalence was 5%, but HCV RNA was detected in only 3.6% of seropositives (overall prevalence 0.18%). Logistic regression for anti-HCV showed poor predictive value (AUC 0.546, *p* = 0.68).

**Conclusion:**

Despite vaccination efforts, a high rate of HBV exposure and waning seroprotection persist among HCWs. The detection of OBI and active HCV in “low-risk” personnel highlights the limitations of current HBsAg-only screening. Epidemiological surveillance should transition to active monitoring of anti-HBc / anti-HBs status and targeted PCR screening, alongside strict adherence to standard precautions by all staff.

## Introduction

In the era of active implementation of the World Health Organization (WHO) global strategies to eliminate viral hepatitis B (HBV) and C (HCV) as public health threats by 2030, studying the epidemiological characteristics in high-burden regions remains a critical task (1). Implementing the WHO strategy requires not only universal coverage of hepatitis B vaccination and treatment but also active epidemiological surveillance in key risk groups (2). According to a leading expert group on hepatitis B and C control, in 2019, 30.4 million people living with hepatitis B were aware of their infection status. In the same year, 15.2 million people were newly diagnosed with hepatitis C. According to various estimates, between 580,000 and 2.4 million people in the United States are living with HBV infection (3). In comparison, in the Netherlands, the prevalence of hepatitis B is estimated at 0.3% (40,000 individuals) and that of HCV at 0.2% (28,000 individuals) (4). In Asian countries, the prevalence varies from low (2–4% in Japan) to moderately high (6% in Pakistan; 15–20% in parts of Southeast Asia) (5, 6). According to official Russian statistics, from 2021 to the present, there has been an increase in the incidence of chronic hepatitis B: from 21 cases per 100,000 population in 2021 to 50 cases per 100,000 in 2025 (7). A similar trend was observed for chronic hepatitis C over the same observation period: from 0.59 cases per 100,000 in 2021 to 0.94 cases per 100,000 in 2025 (7). The North Caucasus region is no exception; in particular, in the Republic of Dagestan, the peak incidence rate of HBV infection during the 2019–2023 observation period was registered in 2019 at 8.78 per 100,000 population. The incidence of HBV infection in 2023 was 7.7 per 100,000 population, which is 12.8% higher than the rate in 2022 (6.73 per 100,000 population) (8, 9). For HCV infection, the incidence rate of chronic forms in 2023 was 8.20 per 100,000 population, representing a 22.8% increase compared to 2022 (6.32 per 100,000 population) (8, 9). For hepatitis C, this rise in incidence can be attributed to the ongoing National Project for the Control of Hepatitis C in Russia, active until 2030, which funds widespread screening (10). At the same time, there is no mass screening program for hepatitis B virus in Russia (11). Healthcare workers (HCWs) and auxiliary staff in healthcare facilities face a continuous occupational risk of infection with bloodborne pathogens. While performing their daily duties – ranging from invasive surgical procedures and blood sampling to medical waste disposal – staff are routinely exposed to hazards associated with cuts, contaminated needlestick injuries, and contact of infected biological fluids with mucous membranes and damaged skin. The risk of viral transmission from a single needlestick injury involving contaminated blood reaches up to 30% for hepatitis B and approximately 3% for hepatitis C (12). The primary tool for the specific prevention of hepatitis B is vaccination using recombinant hepatitis B surface antigen (HBsAg). The mass rollout of this vaccine in many countries, including Russia, has led to a significant decrease in the incidence of acute forms of the infection (13). However, in occupational risk groups, the long-term efficacy of vaccination is challenged by the waning of post-vaccination immunity (14, 15). Although an antibody titer against the hepatitis B surface antigen (anti-HBs) ≥ 10 mIU/mL is widely accepted as the minimum threshold for seroprotection, clinical practice indicates that the concentration of protective antibodies progressively declines over time (16). OBI poses an additional challenge for epidemiological control. OBI is characterized by the persistence of replication-competent HBV DNA in hepatocytes and/or blood despite consistently negative testing results for HBsAg (17, 18). Individuals with occult forms of the infection remain undetected during standard screening programs that focus solely on HBsAg, yet they can still serve as sources of nosocomial viral transmission. Anti-HBc indicates previous or current HBV exposure and may help identify individuals for further evaluation; however, it does not establish a diagnosis of OBI, and its absence does not exclude OBI (18). Regarding hepatitis C, the situation is complicated by the complete absence of a vaccine. The primary preventive measures remain adherence to universal infection control precautions and the use of modern direct-acting antivirals (19, 20). Nevertheless, successful treatment or spontaneous viral clearance leaves specific antibodies in the body.

Primary objective: To determine the seroprevalence of HBV exposure markers (anti-HBc IgG), HCV infection markers (anti-HCV), and the proportion of HCWs with anti-HBs levels ≥10 mIU/mL.

Secondary objectives: (1) To detect occult HBV infection and active HCV infection among seropositive individuals using PCR; (2) To identify independent predictors of anti-HBc IgG positivity using multivariable logistic regression; (3) To assess the association between anti-HBs antibody levels and anti-HBc positivity.

## Materials and methods

### Study design and participants

A cross-sectional observational study was conducted. The study enrolled 1,643 HCWs and auxiliary staff from healthcare facilities in a region with moderate-to-high endemicity for HBV and HCV. All participants provided written voluntary informed consent to participate in the study.

### Inclusion and exclusion criteria

Inclusion criteria:

- Healthcare facility employees aged 18 to 75 years;
- Agreement to comply with the study protocol requirements;
- Documented history of vaccination against HBV.

Exclusion (non-inclusion) criteria:

- A verified diagnosis of chronic viral hepatitis B, D, or C;
- Acute viral or bacterial infectious diseases, or exacerbation of chronic diseases at the time of examination;
- Verified HIV infection;
- Lack of documented information on prophylactic vaccination against HBV;
- Vaccination against HBV due to epidemic indications (emergency vaccination);
- History of blood or blood component transfusion within the past 12 months;
- History of blood or blood component donation, or invasive dental, tattooing, or other procedures within 3 months prior to blood collection;
- Use of immunosuppressive drugs or immunoglobulins within the preceding 12 months;
- Presence of oncological diseases.

Individuals with a previously verified diagnosis of chronic viral hepatitis B, C, or D were excluded because the primary aim was to detect undiagnosed (occult or latent) infections among ostensibly healthy HCWs. Individuals with a previously diagnosed chronic HBV, HCV, or HDV infection were excluded because the study focused on screening findings among employees without a known diagnosis of chronic viral hepatitis. The reported estimates therefore apply to the eligible study population and should not be interpreted as the overall prevalence among all employees. Participants were included regardless of the time elapsed since their last HBV vaccination dose. The interval between the last documented vaccination and blood sampling was not used as an inclusion / exclusion criterion, as one of the study objectives was to assess the real-world status of post-vaccination immunity across different time intervals.

The study was approved by the Local Ethics Committee of I. M. Sechenov First Moscow State Medical University (Sechenov University), Moscow, Russian Federation (protocol No. 1323, approved on July 20, 2023). The ethics approval covered: (a) serological testing of blood samples; (b) storage of serum aliquots at −70 °C; and (c) subsequent molecular testing of seropositive samples using stored aliquots. All eligible employees at the participating facilities were invited to take part. The final sample comprised employees who were present during the screening sessions and consented to participation; it was therefore not a probability sample. Participants were individually informed of their personal screening results, including PCR findings, at the conclusion of the laboratory phase. Participants with positive results were referred for clinical evaluation. The sampling frame comprised all healthcare workers and auxiliary staff employed at multidisciplinary healthcare facilities during the study period July, 2023 – May, 2025. The study was conducted at several multidisciplinary hospitals in the Republic of Dagestan. The participating institutions provide specialized inpatient and outpatient care in fields such as internal medicine, surgery, pediatrics, and laboratory medicine. Participants were recruited from among the staff of clinical, surgical, laboratory, administrative, and support departments at these institutions. Recruitment followed a census-based approach: all eligible employees present during the designated screening periods were invited to participate. Participation was voluntary; those who declined or were absent during the screening period were not included. Demographic data, including age, sex, and other details, were obtained through structured interviews with the study participants. Participants’ personal data were de-identified. Laboratory results were collected and stored using individual research ID numbers in a secure information system. The key linking the research ID to the participant’s identity was stored separately and was accessible only to the researcher responsible for individually informing participants of their test results. Upon completion of the laboratory phase, each participant was individually notified of their personal screening results.

Additionally, all participants were classified into three categories of occupational risk for bloodborne viral infections based on the nature of their work:

- High risk: Daily contact with blood and bodily fluids, frequent use of surgical instruments, high likelihood of sharp injuries, and work with high-risk patient populations;
- Medium risk: Regular but less frequent contact with bodily fluids, episodic performance of invasive procedures, and ward patient care;
- Low risk: Minimal contact with blood and bodily fluids, predominantly advisory or administrative activities without invasive procedures, as well as auxiliary staff.

Occupational risk categorization was performed by the study investigators based on each participant’s job title and corresponding occupational duties, following the principles outlined in the Russian national sanitary regulations and adapted from international frameworks (10, 11). Classification was based on job title and the typical nature of duties associated with each position, rather than individual-level exposure assessment. Individual-level data on needlestick injuries or sharps exposures were not collected in this study, which is acknowledged as a limitation.

For the purposes of this study, healthcare workers were defined as physicians, nursing staff (mid-level medical professionals), and nursing assistants (junior medical staff) who provide direct patient care. Auxiliary staff comprised non-medical personnel (administrative, cleaning, laundry, catering, and technical support staff) employed at healthcare facilities who may have incidental contact with biological materials or contaminated environments. The sample size was estimated *a priori* using the formula for cross-sectional prevalence studies: *n* = *Z*^2^ × *p* × (1 − *p*)/*d*^2^, where *Z* = 1.96 (95% confidence level), *p* = 0.20 (estimated anti-HBc prevalence among HCWs based on prior regional data), and *d* = 0.02 (desired precision of ±2%). The minimum required sample size was calculated as 1,537. To account for potential incomplete data or refusals, recruitment targeted a minimum of 1,600 participants. The final sample of 1,643 exceeded this requirement.

*Sample collection and laboratory analysis.* Blood samples were collected from participants in disposable plastic tubes containing a clot activator. The samples were allowed to clot at room temperature for 15 min, then centrifuged at 3000 rpm for 10 min to separate the serum. Serum aliquots were stored at −70 °C for no more than 4 weeks prior to analysis. Testing of patient specimens was performed at the Laboratory of Vaccine Prevention and Immunotherapy of Allergic Diseases, Mechnikov Research Institute of Vaccines and Sera, using certified equipment from the institute’s shared facility. All participants were tested for HBsAg using the “Vector-Best HBsAg,” Russia, with analytical sensitivity of 0.05 IU/mL. Testing was performed on serum samples collected during the initial screening. HBsAg-positive individuals were identified and referred for clinical follow-up. HBV DNA testing was performed only in HBsAg-negative, anti-HBc-positive participants. Quantitative determination of antibodies to the hepatitis B surface antigen (anti-HBs) was performed by enzyme-linked immunosorbent assay (ELISA) using the “Vector-Best anti-HBs-IgG” test system (Russia) in accordance with the manufacturer’s instructions (21). An anti-HBs level ≥10 mIU/mL was used as the conventional serological threshold for seroprotection, in accordance with international guidelines. A higher threshold of ≥100 mIU/mL was applied as an exploratory higher-titer category, as suggested by some authors for occupational risk groups, but is not an established criterion for defining susceptibility (17, 18). The level of antibodies to the hepatitis B core antigen (anti-HBc IgG) was determined by ELISA using the “GepaBest anti-HBc-IgG” test system (JSC “Vector-Best-Europe,” Russia) (14). Total antibodies to the structural (Core) and non-structural (NS) proteins of hepatitis C virus (anti-HCV core, anti-HCV NS) were determined by ELISA using the “Hepatitis IFA-anti-HCV-Spectrum” test system (LLC “Alkor Bio,” Russia) (22). All assays were performed in strict compliance with the manufacturers’ instructions. Viral DNA/RNA testing was performed at the laboratory of Molecular Epidemiology of Viral Hepatitis of the Central Research Institute of Epidemiology of Rospotrebnadzor, Russia. Serum samples positive for anti-HBc IgG were analyzed for qualitative detection of HBV DNA by real-time polymerase chain reaction (PCR) using the AmpliSens HBV-FL reagent kit according to the manufacturer’s instructions (23). The serum volume used for extraction was 1,000 μL; the detection limit of the kit was 10 IU/mL. Serum samples positive for total anti-HCV core and anti-HCV NS antibodies were tested for HCV RNA using reverse transcription followed by real-time PCR with the AmpliSens HCV-FL reagent kit in accordance with the manufacturer’s instructions (23).

Statistical data processing was performed using R version 4.5.3 (R Core Team, R Foundation for Statistical Computing, Vienna, Austria) and jamovi version 2.7.38 (jamovi open-source software, Australia). Categorical variables are presented as absolute numbers and percentages; differences between groups were assessed using Pearson’s *χ*2 test or Fisher’s exact test (when expected frequencies were <5 in any cell of the contingency table). Quantitative variables with a non-normal distribution (assessed using the Shapiro – Wilk test) are presented as median and interquartile range (IQR). The Mann – Whitney *U* test was used to compare two independent groups, and the Kruskal–Wallis test with Holm’s correction was used for post-hoc pairwise comparisons of more than two groups. Multivariable binary logistic regression models were used to identify independent predictors of anti-HBc and anti-HCV seropositivity. Variable selection was guided by clinical relevance and univariable screening (*p* < 0.10). Reference categories were predefined (e.g., lowest risk groups, male sex, younger age brackets). Multicollinearity among covariates was evaluated using variance inflation factors. Model fit and goodness-of-fit were assessed via the likelihood ratio test (*χ*^2^), Nagelkerke pseudo-*R*^2^, and the area under the receiver operating characteristic curve (AUC). Age was evaluated both continuously and categorically to assess non-linear effects. Missing data were minimal (<1%) and handled via complete-case analysis. Additionally, a sensitivity analysis was conducted using log10-transformed continuous anti-HBs values, which corroborated the findings obtained with categorical thresholds. Results are presented as adjusted odds ratios (aOR) with 95% confidence intervals (95% CI). Statistical significance for all tests was set at *p* < 0.05.

## Results

### General characteristics of the study population

The total study population comprised 1,643 individuals. The median age of the participants was 48 years (IQR 36–59). The majority of HCWs and auxiliary staff were female, accounting for 1,519 (92.5%) individuals. The distribution of participants by occupational categories in the healthcare facilities was as follows (Table 1): 25.8% were physicians, 49.4% were nursing staff (mid-level medical staff), 18.8% were junior medical staff, and 6% represented other personnel (catering, administrative, and maintenance staff, etc.). Additionally, all HCWs were stratified by their occupational risk level for potential viral hepatitis infection into three main categories: high risk, medium risk, and low risk (Table 1).

**Table 1:** Baseline demographic and occupational characteristics of the study participants.

Characteristic	n (%)
Staff category	
Female	1,519 (92.5)
Male	124 (7.5)
Age, median (IQR)	48 (IQR 36–59)
Physicians	425 (25.8)
Nurses	811 (49.4)
Nursing assistants	308 (18.8)
Other staff	99 (6)
Occupational risk group	
High risk (daily contact with blood/body fluids Frequent use of sharp instruments; high probability of needlestick injuries; work with high-risk patients)	558 (34)
Medium risk (Regular but less frequent exposure; occasional invasive procedures; ward-based patient care)	201 (12.3)
Low risk (Minimal blood/body fluid contact; primarily consultative or administrative duties; no invasive procedures)	884 (53.8)

As shown in Table 1, 34.0 and 12.3% of the study participants had high and medium occupational risks of infection, respectively, due to the nature of their professional activities. These groups predominantly consisted of healthcare workers with direct exposure to blood and bodily fluids (e.g., blood, semen, and cerebrospinal fluid) – such as surgeons, obstetricians-gynecologists, midwives, and procedural nurses – or those with close patient contact, including dentists, dental assistants, and infectious disease specialists.

### Anti-HBs antibody levels in healthcare workers

At the time of enrollment, only 901 (54.8%) participants had anti-HBs levels ≥10 mIU/mL. Notably, a high antibody level (≥ 100 mIU/mL) was detected in only 488 (29.7%) individuals.

We observed no significant age-related differences between participants with and without anti-HBs levels ≥10 mIU/mL (*p* = 0.926).

We analyzed the post-vaccination anti-HBs antibody titers among HCWs and auxiliary staff (Figure 1A). For this analysis, antibody titers were evaluated based on the participants’ occupational categories: Group 1, physicians; Group 2, nursing staff (mid-level medical staff); Group 3, junior medical staff; and Group 4, other (auxiliary) personnel. No significant intergroup differences were identified within the study population (*p* = 0.137). The median anti-HBs antibody concentration among physicians was 15.3 mIU/mL (IQR 0.34–151). Among junior medical staff, the median concentration was the lowest, at 9.85 mIU/mL (IQR 0.01–102). The highest median concentration was observed among nursing staff at 21 mIU/mL (IQR 1.42–180). The median concentration for auxiliary personnel was 15.8 mIU/mL (IQR 2.29–177). When comparing the proportion of individuals with anti-HBs levels in the higher-titer category (≥100 mIU/mL) across these occupational categories, no statistically significant differences were found (*p* = 0.310) (Figure 2B). Specifically, the proportion of physicians with anti-HBs ≥ 100 mIU/mL was 29% (Figure 1B).

**Figure 1:**
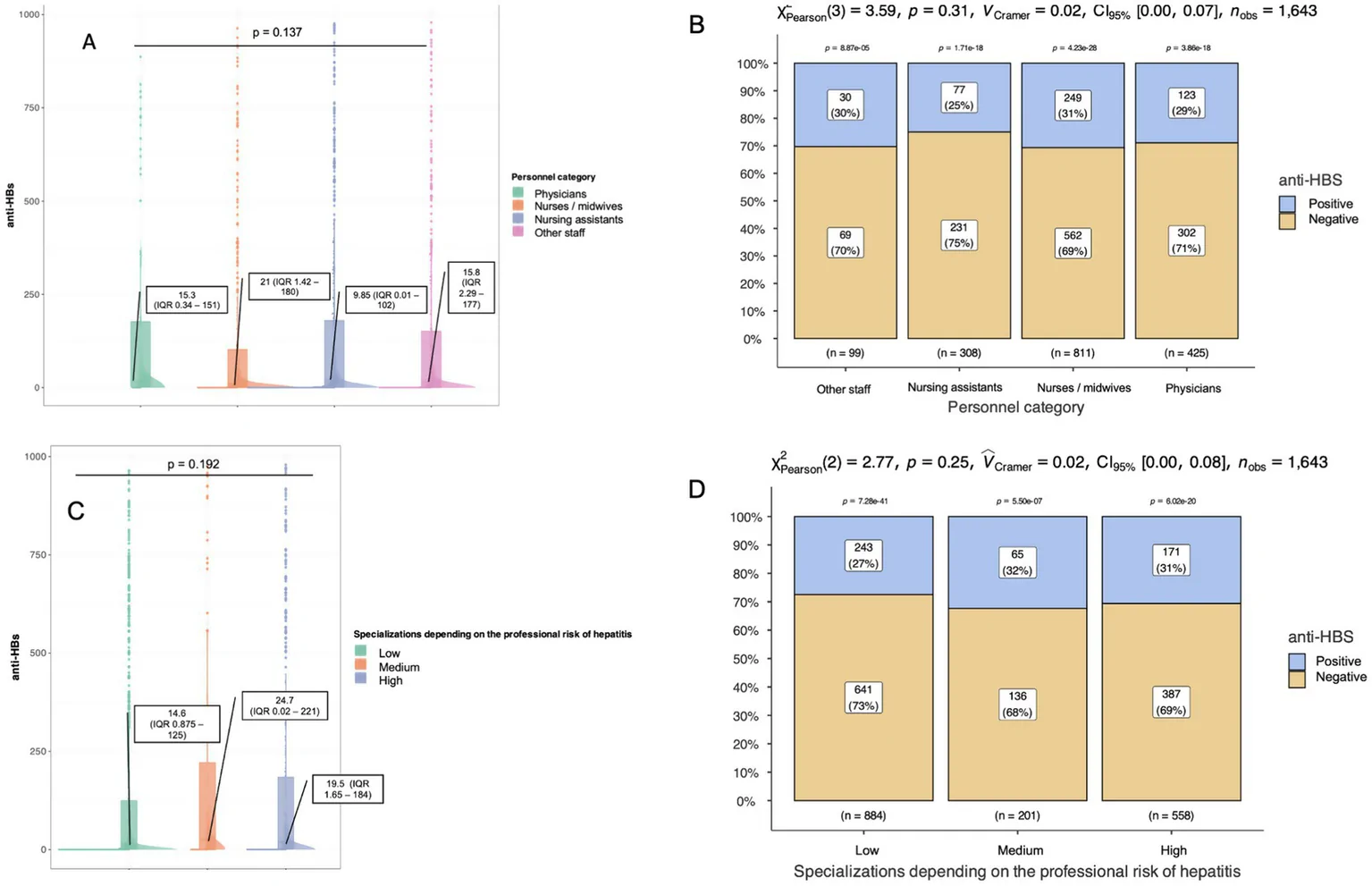
Median concentrations and proportions of seroprotective anti-HBs antibody titers among individuals based on occupational category and risk of infection (*n* = 1,643). Quantitative antibody data across groups are presented as medians with interquartile ranges (the Kruskal – Wallis test was used for intergroup comparison); qualitative data are presented as proportions (Pearson’s *χ*^2^ test was used for intergroup comparison); *p* < 0.05 was considered statistically significant.

**Figure 2:**
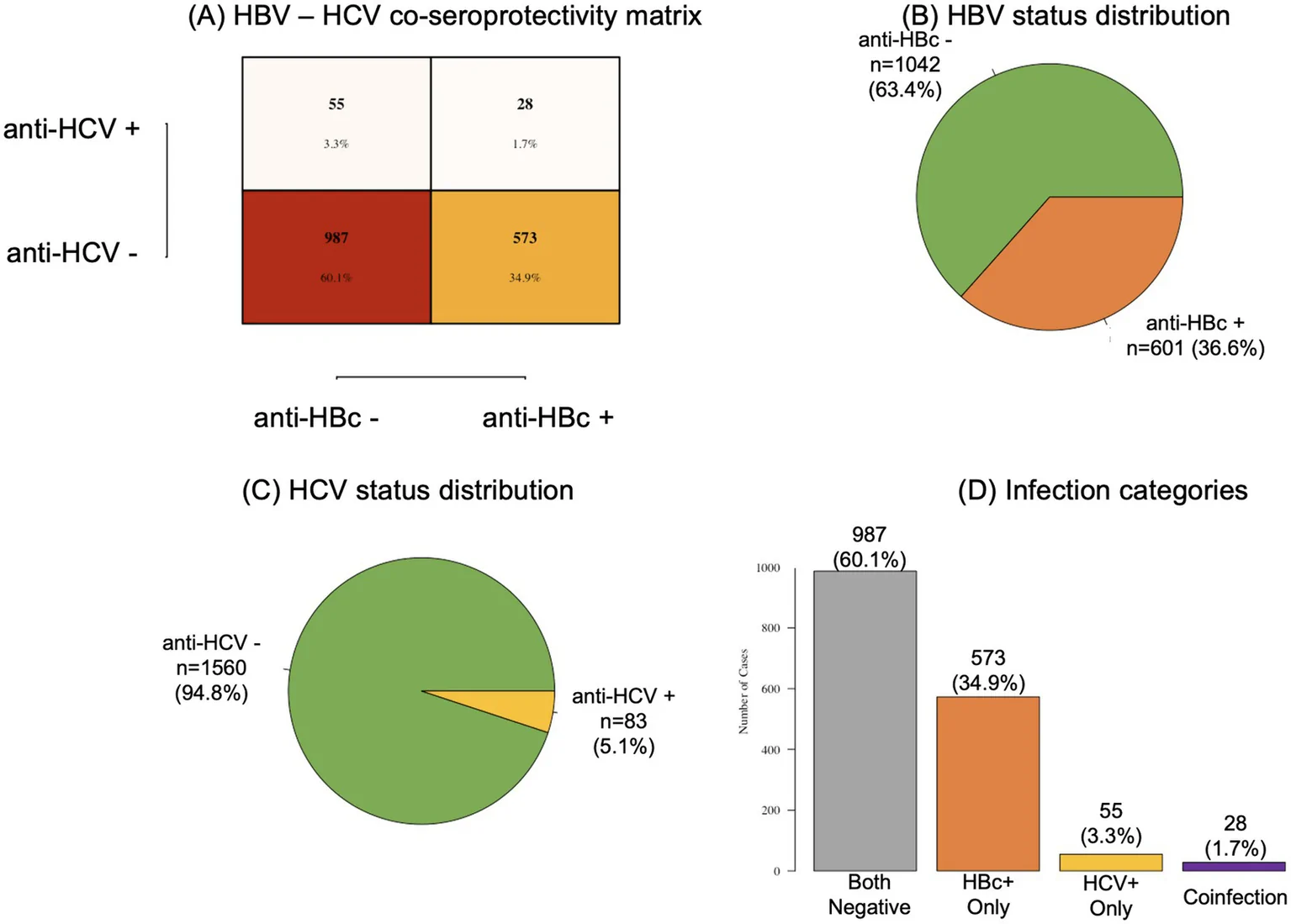
Heatmap of the distribution of serological status for HBV and HCV infections in the study participants. **(A)** Two-by-two matrix heatmap assessing serostatus for anti-HBc and anti-HCV antibodies; pie charts illustrating the distribution of serological status for HBV **(B)** and HCV **(C)** infections; **(D)** Grouped bar chart showing the distribution across four serological categories.

Additionally, we evaluated anti-HBs antibody concentrations in relation to the occupational risk of potential HBV infection (Figure 1C). We found no significant differences based on the level of occupational risk (*p* = 0.192). The median anti-HBs antibody concentration in the high-risk group was 19.5 mIU/mL (IQR 1.65–184). The highest median concentration was observed in the medium-risk group, reaching 24.7 mIU/mL (IQR 0.02–221). The concentration of anti-HBs antibodies in the low-risk group was 14.6 mIU/mL (IQR 0.875–125). An intergroup comparison of the proportion of individuals with anti-HBs ≥ 100 mIU/mL did not differ significantly (*p* = 0.250) (Figure 1D). In the high- and medium-risk groups, anti-HBs ≥ 100 mIU/mL was observed in 31 and 32% of individuals, respectively (Figure 1D).

### Prevalence of HBV infection markers

During the primary serological screening, 601 (36.6%) healthcare workers were newly identified as positive for anti-HBc IgG antibodies in the absence of HBsAg. We found that anti-HBc IgG-positive individuals were significantly older (Me 53 years IQR 43–61) than their antibody-negative counterparts (Me 42 years IQR 33–56) (*p* < 0.001).

A total of 252 (41.9%) individuals had anti-HBs titers ≥10 mIU/mL together with anti-HBc IgG positivity. Notably, only 33.3% of anti-HBc IgG-positive individuals were associated with an increased occupational risk of HBV infection, whereas the majority (57.2%) belonged to the low occupational risk group. However, no statistically significant differences were observed between these groups.

### Diagnosis of occult hepatitis B infection

Final PCR testing for the presence of HBV DNA was performed on 129 healthcare workers from a single facility. Based on real-time PCR results, HBV DNA was detected in 4 (3.1%; 95% CI 0.85–7.73%) HCWs who were HBsAg-negative and anti-HBc-positive. The median age of these individuals was 56.5 years (IQR 51.5–59.8), and 75% were female. One of the workers with a positive PCR test belonged to the high occupational risk group for viral hepatitis, while the remaining individuals were in the low-risk category. A notable finding was the significantly lower concentration of anti-HBs antibodies in patients with occult HBV infection compared to those with a negative DNA test (Me 7 mIU/mL (IQR 0–15) vs. 165 mIU/mL (IQR 55–480); *p* = 0.003).

### Diagnosis of HCV infection

Additionally, blood serum samples were screened for markers of HCV infection. Out of the 1,643 samples tested, total antibodies to structural (anti-HCV core) and non-structural (anti-HCV NS) proteins of hepatitis C virus were newly detected in 83 (5.0%) healthcare workers (Figure 1). The median age of individuals positive for HCV infection markers was 47 years (IQR 32.5–57) (Table 2).

**Table 2:** Demographic and occupational characteristics of individuals with positive and negative status for anti-HCV antibodies.

Parameter	Anti-HBc-positive (*n* = 601)	Anti-HBc-negative (*n* = 1,042)	cOR; 95% Cl	*p*-value	aOR; 95% Cl	*p*-value
Age (years), median (IQR)	46 (32–57)	48 (36–59)	n/a	0.210	n/a	0.210
Per 10-year increment			0.89 (0.76–1.05)	0.179	0.89 (0.75–1.06)	0.181
Sex						
Female (ref.)	76 (92.7%)	1,443 (92.4%)	n/a	n/a	n/a	n/a
Male	6 (7.3%)	118 (7.6%)	0.97 (0.41–2.26)	0.936	0.98 (0.41–2.33)	0.958
Anti-HBc IgG status						
Negative (ref.)	54 (65.9%)	988 (63.3%)	n/a	n/a	n/a	n/a
Positive	28 (34.1%)	573 (36.7%)	0.89 (0.56–1.43)	0.639	0.98 (0.60–1.59)	0.927
Occupational risk category (ref. High)						
High	26 (31.7%)	532 (34.1%)	n/a	n/a	n/a	n/a
Medium	11 (13.4%)	190 (12.2%)	1.18 (0.57–2.44)	0.647	1.20 (0.57–2.49)	0.633
Low	45 (54.9%)	839 (53.7%)	1.10 (0.67–1.80)	0.713	1.17 (0.70–1.95)	0.550

Age entered as continuous variable (per 10-year increment) in the logistic regression model. cOR, crude odds ratio (univariable logistic regression); aOR, adjusted odds ratio (multivariable logistic regression); 95% CI, 95% confidence interval; IQR, interquartile range; Ref., reference category. Me (IQR), median (interquartile range); n/a, no data available.

Similar to the findings for HBV, a slightly higher number of anti-HCV-positive individuals were associated with a low occupational risk. However, this difference was not statistically significant (*p* = 0.863). Notably, 33.7% of anti-HCV-positive individuals were also positive for anti-HBc antibodies. The distribution of the four serological profiles of anti-HCV and anti-HBc antibodies within the study participants is shown in Figure 2.

As shown in Figures 3A, 987 (60.1%) study participants were seronegative for both anti-HCV and anti-HBc antibodies. A total of 573 (34.9%) individuals were positive only for anti-HBc antibodies but negative for anti-HCV. The converse status (presence of anti-HCV antibodies in the absence of anti-HBc) was observed in 55 (3.3%) subjects (Figure 2).

**Figure 3:**
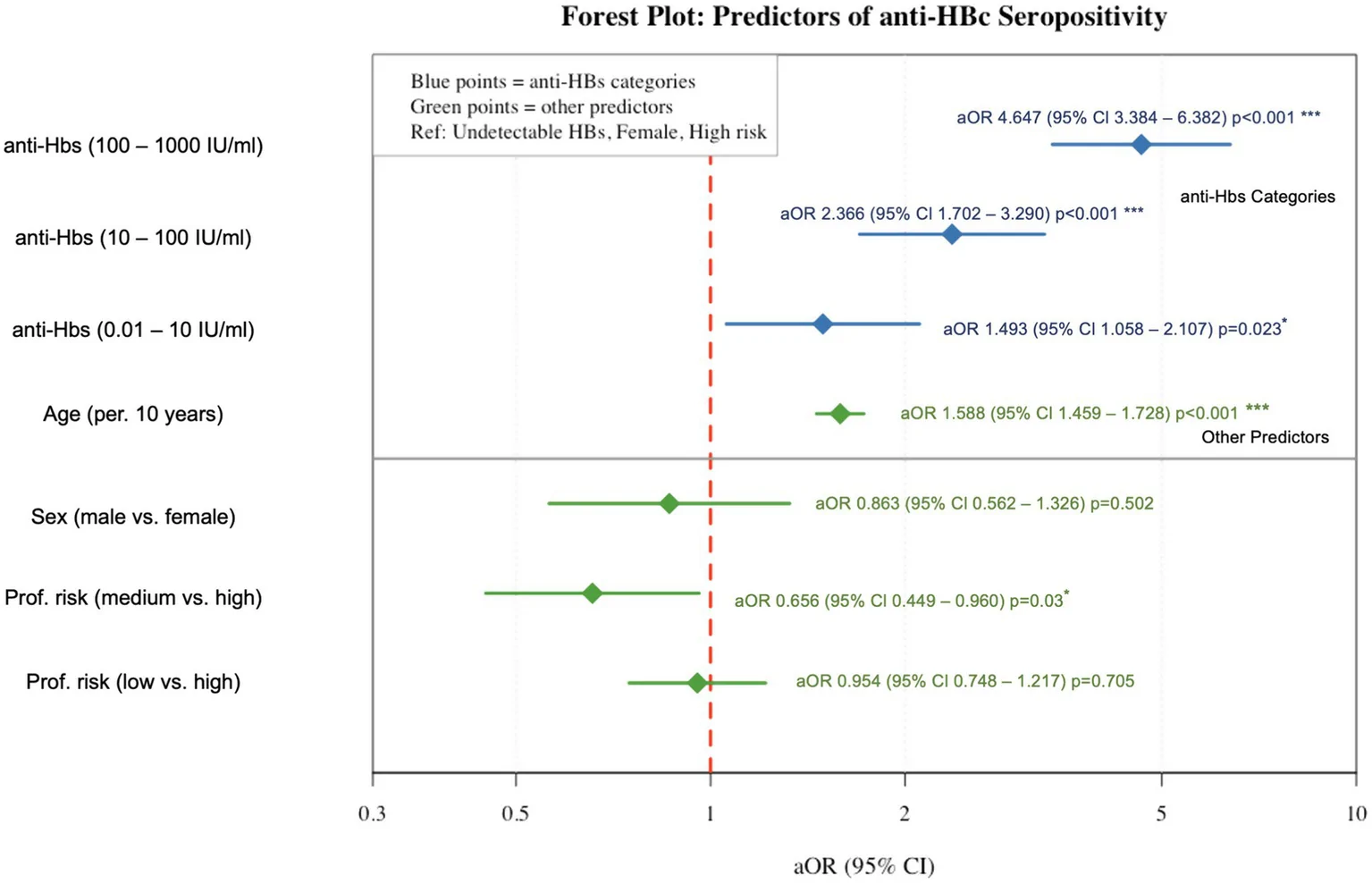
Logistic regression model of predictors for anti-HBc IgG positivity. Multivariable logistic regression model showing adjusted odds ratios (aORs) and 95% confidence intervals (95% CIs) for independent predictors of anti-HBc IgG seropositivity; *p* < 0.05 was considered statistically significant.

Additionally, all anti-HCV-positive samples (*n* = 83) were analyzed by PCR, which detected HCV RNA in 3 (3.6%) individuals. This group comprised two males and one female, all of whom belonged to the low occupational risk category. Notably, of the three individuals with active HCV infection, two were also positive for anti-HBc IgG antibodies. However, HBV DNA PCR was negative in both of these individuals.

### Predictors of anti-HBc IgG positivity in healthcare workers

To identify independent predictors of a positive anti-HBc IgG test result in the study participants, a multivariable logistic regression model was constructed (Figure 3). The model demonstrated good discriminatory ability (AUC = 0.722) and was statistically superior to the null model (*χ*^2^ 236.6, df 7, *p* < 0.001). Logistic regression analysis demonstrated a clear monotonic increase in the odds of anti-HBc antibody positivity with increasing levels of anti-HBs antibodies, after adjusting for age, sex, and occupational risk (Figure 3). For anti-HBs antibody titers ranging from 0.01 to 10 mIU/mL, the odds of anti-HBc antibody presence were 1.5 times higher (*p* = 0.023) compared to the reference group. For titers between 10 and 100 mIU/mL, the odds increased 2.4-fold (*p* < 0.001) (Table 3). This effect was most pronounced at high anti-HBs antibody titers; individuals with anti-HBs antibody levels between 100 and 1,000 mIU/mL had 4.65-fold higher odds of testing positive for anti-HBc IgG compared to those with undetectable anti-HBs antibodies (95% CI: 3.38–6.38; *p* < 0.001) (Table 3). Age remained a significant independent predictor. For each year of age, the odds of anti-HBc antibody presence increased by 4.7% (*p* < 0.001). For every 10 years of age, this cumulative effect resulted in a 1.55-fold increase in the odds of a positive anti-HBc IgG test result (*p* < 0.001). Sex did not significantly affect the likelihood of positive anti-HBc antibody status (*p* = 0.502), which we attribute to the highly skewed distribution of this parameter in our study population. Occupational risk stratification revealed that healthcare workers in medium-risk departments had significantly lower odds of testing positive for anti-HBc IgG compared to those in high-risk departments (aOR 0.66, *p* = 0.03) (Table 3).

**Table 3:** Demographic and occupational characteristics of individuals with positive and negative anti-HBc IgG status, and logistic regression results.

Parameter	Anti-HBc-positive (*n* = 601)	Anti-HBc-negative (*n* = 1,042)	cOR; 95% Cl	*p*-value	aOR; 95% Cl	*p*-value
Age (years), median (IQR)	53 (43–61)	42 (33–56)	n/a	<0.001*	n/a	<0.001^*^
Per 10-year increment			1.52 (1.41–1.65)	<0.001^*^	1.59 (1.46–1.73)	<0.001^*^
Sex						
Female (ref.)	561 (93.3%)	958 (92.0%)	n/a	n/a	n/a	n/a
Male	40 (6.7%)	84 (8.0%)	0.81 (0.55–1.20)	0.299	0.86 (0.56–1.33)	0.503
Occupational risk category (ref. High)						
High	200 (33.3%)	358 (34.4%)	n/a	n/a	n/a	n/a
Medium	57 (9.5%)	144 (13.8%)	0.71 (0.50–1.01)	0.055	0.66 (0.45–0.96)	0.030^*^
Low	344 (57.2%)	540 (51.8%)	1.14 (0.92–1.42)	0.241	0.95 (0.75–1.22)	0.709
Anti-HBs titer category, mIU/mL (ref. undetectable, 0)						
Undetectable	127 (21.1%)	246 (23.6%)	n/a	n/a	n/a	n/a
0.01–10	146 (24.3%)	224 (21.5%)	1.38 (0.99–1.92)	0.056	1.49 (1.05–2.10)	0.024*
10–100	141 (23.5%)	271 (26.0%)	2.01 (1.47–2.76)	<0.001^*^	2.38 (1.71–3.31)	<0.001^*^
100–1,000	187 (31.1%)	301 (28.9%)	3.89 (2.88–5.26)	<0.001^*^	4.65 (3.38–6.38)	<0.001^*^

Age entered as continuous variable (per 10-year increment) in the logistic regression model. cOR, crude odds ratio (univariable logistic regression); aOR, adjusted odds ratio (multivariable logistic regression); 95% CI, 95% confidence interval; IQR, interquartile range; Ref., reference category. Me (IQR), median (interquartile range); n/a, no data available. *p* < 0.05 is considered statistically significant. Significant results are marked with an asterisk (^*^).

### Predictors of total anti-HCV positivity in healthcare workers

The multivariable logistic regression model for anti-HCV seropositivity yielded an AUC of 0.546 (95% CI 0.44–0.65; *p* = 0.68), indicating no meaningful discriminative ability beyond chance (Figure 4). No independent predictor reached statistical significance after adjustment. These findings should be interpreted cautiously: the model may be underpowered given the relatively small number of seropositive cases (*n* = 82), and the absence of a significant predictor does not establish that risk is uniformly distributed (Table 2). Larger, purpose-designed studies are needed to identify reliable predictors of HCV exposure in healthcare workers.

**Figure 4:**
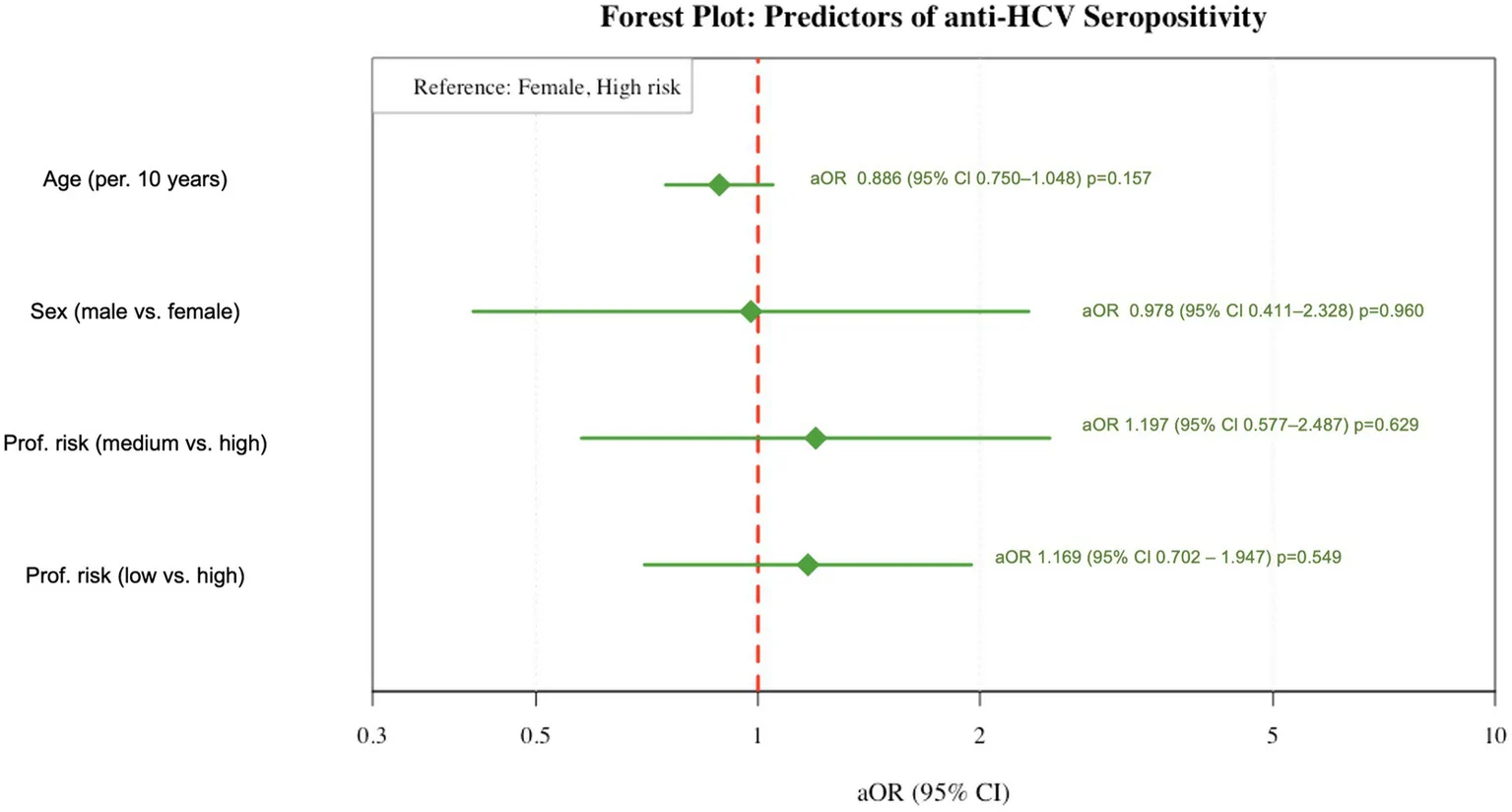
Logistic regression model of predictors for anti-HCV antibody positivity. Multivariable logistic regression model showing adjusted odds ratios (aORs) and 95% confidence intervals (95% CIs) for independent predictors of antibody seropositivity; *p* < 0.05 was considered statistically significant.

### Age-specific analysis of HBV and HCV infection

To evaluate seroprotection and the prevalence of viral antibodies among HCWs across different age groups, we analyzed age-related trends using logistic regression (Figure 5). We found that anti-HBc antibody seropositivity demonstrated a pronounced age dependence (Figure 5A). The prevalence gradually increased from 12.4% (17/137) among workers younger than 30 years to 50.0% (213/426) among those older than 60 years (aOR = 1.55 per 10 years of age; *p* < 0.001). Workers younger than 40 years exhibited a significantly lower prevalence of anti-HBc antibodies (15.5%) compared to those older than 50 years (46.4%), representing a 67% relative reduction in the likelihood of HBV infection (*χ*^2^ 51.4; *p* < 0.001) (Figure 5A). In contrast, HBV seroprotection rates remained relatively stable across all age groups, ranging from 51.9 to 59.9% (*p* = 0.118) (Figure 5B).

**Figure 5:**
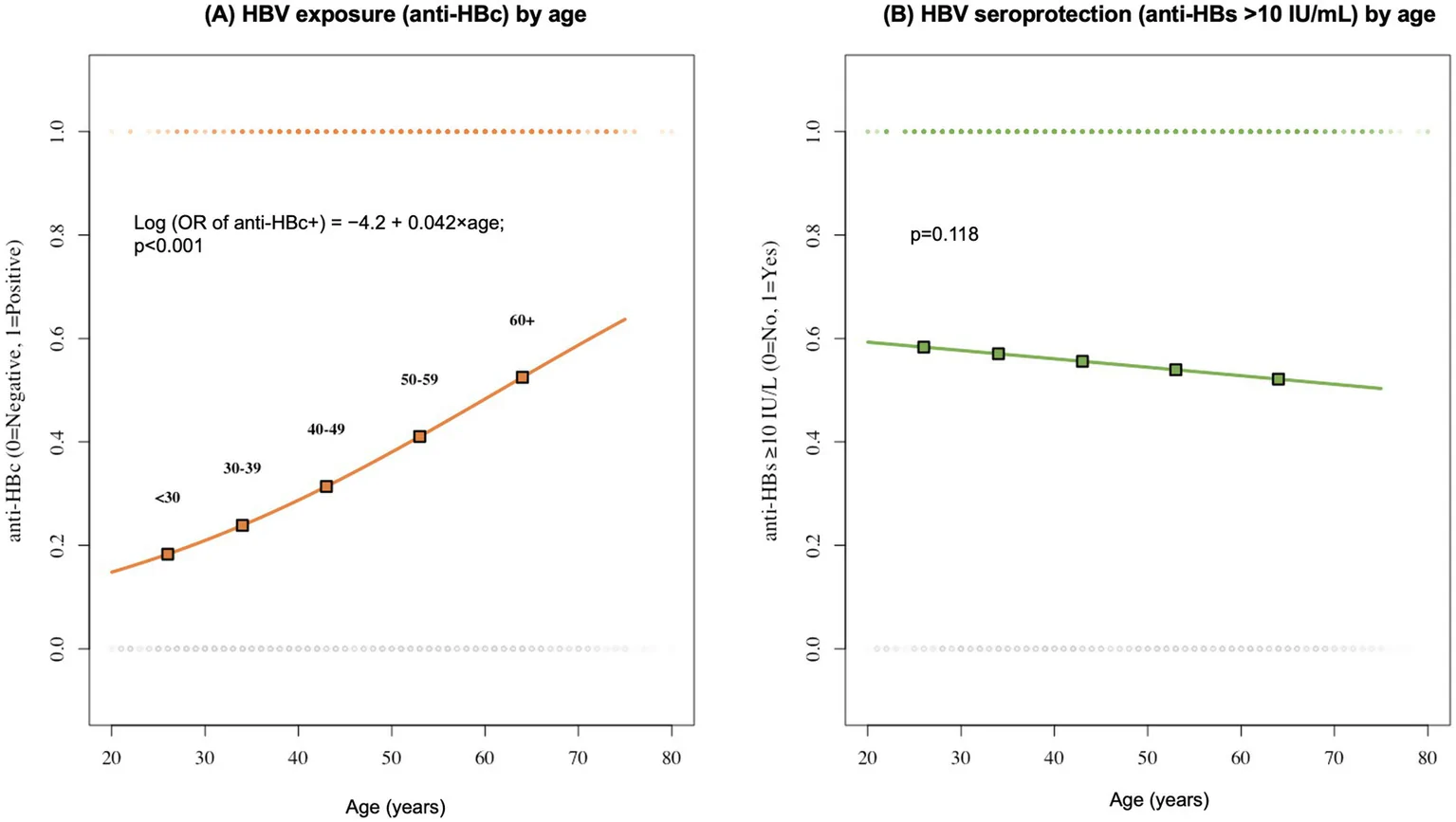
Prevalence of anti-HBc **(A)** and anti-HBs **(B)** antibodies across different age groups. Scatter plot with a logistic regression curve illustrating antibody seropositivity (logistic regression analysis showing odds ratios; *p* < 0.05 was considered statistically significant).

## Discussion

The present study provides a cross-sectional epidemiological analysis of the prevalence of viral HBV and HCV infections among HCWs and auxiliary staff at multidisciplinary healthcare facilities. These findings are of clinical and scientific relevance. Analysis of official epidemiological data in Russia for 2025 reveals a pronounced divergent trend: alongside declining incidence rates of acute viral hepatitis, high prevalence levels of chronic forms persist (7). On the one hand, HCWs exhibit high vaccination coverage in the younger age groups (aligned with WHO targets); on the other hand, they may harbor undetected HBV infection, as evidenced by the detection of HBV DNA in 4 of 129 anti-HBc-positive HCWs. While these findings raise the theoretical concern of potential transmission risk, no direct evidence of occupational transmission was observed in this study, and the clinical significance of low-level HBV DNA detection remains to be established. For comparison, new guidelines for HBV screening have been in place in the United States since 2023 (24). This screening involves testing for HBsAg, total anti-HBc, and anti-HBs antibodies. Furthermore, screening is recommended for all individuals aged 18 and older, regardless of prior vaccination history (24). Screening in subgroups with higher observed seroprevalence entails HBsAg and anti-HBc testing alongside PCR diagnostics (25). Similar recommendations are established in New Zealand, Japan, and Europe (25). In Russia, mass screening for hepatitis B is not implemented; mandatory screening is restricted to specific categories of individuals, primarily HCWs, and relies solely on HBsAg detection (11). However, this approach is insufficient, particularly within subgroups with higher observed seroprevalence. The detection of anti-HBc IgG in 36.6% of HCWs indicates a high level of previous or current HBV exposure within this population. This rate substantially exceeds anti-HBc prevalence levels reported among medical staff in Western Europe and North America (ranging from 2 to 7%) but is comparable to data from intermediate- and high-endemicity regions (Asia, and parts of Africa and South America, where rates reach 20–45%) (17, 18). The primary factor explaining this pattern is the pronounced age gradient within the study population. Our regression model demonstrated that the odds of anti-HBc detection increased by 4.7% per year of life. In our study, anti-HBc prevalence was only 12.4% among HCWs under 30 years of age, compared to 50% in those over 60, suggesting that universal childhood HBV vaccination, introduced in Russia in the mid-1990s, has substantially reduced exposure to natural HBV in younger birth cohorts, consistent with WHO elimination targets. We attribute this finding to the historical timeline of universal hepatitis B vaccination implementation in Russia. Mass vaccination against HBV infection was initiated in the mid-1990s (26). Consequently, HCWs under 30–35 years of age entered professional practice having already been vaccinated in childhood, which likely provided protection prior to exposure to occupational hazards. Conversely, HCWs in the older age groups (50–60 years and older) began their careers in the 1980s and 1990s. During this period of high viral hepatitis incidence and in the absence of preventive vaccination, individuals in this age group had a much higher likelihood of exposure to HBV during invasive and other medical procedures (26).

One of the most critical findings of our study is that anti-HBs levels ≥10 mIU/mL were found in only 54.8% of the participants. Although 45.2% of HCWs had anti-HBs levels below 10 mIU/mL, it should be noted that immune memory may persist even when measurable anti-HBs levels decline below the conventional protective threshold. The absence of detectable anti-HBs does not necessarily equate to susceptibility, particularly in previously vaccinated individuals. Moreover, when a higher-titer threshold (≥100 mIU/mL) is applied, the proportion of individuals meeting this criterion drops to 29.7%. This more conservative threshold is dictated by clinical necessity; studies suggest that individuals at high occupational risk may require higher anti-HBs antibody titers (17, 18, 27, 28). In Russia, as well as in the US, Portugal, and Romania, standard hepatitis B vaccination begins at birth using a 0-1-6 month schedule (15, 29, 30). High-risk infants receive an accelerated schedule of 0-1-2-12 months (28, 29). In several EU countries, including Germany, France, Italy, Spain, and Austria, vaccination starts later, at 2–3 months of age, while Denmark, Norway, and Sweden do not have universal schedules, vaccinating only high-risk infants (30). In these countries, subsequent immunization is selective and target-driven. In Russia, vaccination of healthcare workers and other occupational risk groups is mandated once every 10 years without intermediate monitoring of anti-HBs levels (30). In contrast, international organizations, such as the CDC, recommend post-vaccination serological monitoring 2–3 months after completing the primary series to identify non-responders or those with insufficient protective titers (>10 mIU/mL) (31).

A striking paradox identified in our study warrants special consideration: individuals with high anti-HBs titers (100 to 1,000 mIU/mL) had 4.65-fold higher odds of being anti-HBc-positive compared to those with undetectable antibodies (95% CI 3.38–6.38; *p* < 0.001). One possible explanation for this association is the phenomenon of natural boosting, whereby periodic subclinical exposure to HBV in the healthcare environment may stimulate anamnestic anti-HBs responses in previously infected individuals. However, this hypothesis cannot be confirmed by our cross-sectional data, and alternative explanations including differences in primary immune response or vaccination timing cannot be excluded (32). Vaccinated individuals who have not been exposed to the natural HBV infection virus exhibit a faster decline and subsequent loss of protective anti-HBs titers over time (16). Thus, in endemic settings, elevated post-vaccination antibody titers among healthcare personnel may paradoxically serve as an indirect indicator of frequent viral contact rather than solely reflecting vaccine efficacy.

Furthermore, the issue of OBI remains one of the most challenging problems in modern hepatology (17). OBI is characterized by the presence of replicative HBV DNA in liver tissue (and, in some cases, in the blood) in the absence of detectable surface antigen (HBsAg), and can trigger acute hepatitis B when transmitted from a donor to an immunosuppressed recipient (18, 33). In our study, HBV DNA PCR testing was performed on 129 HCWs from a single facility who were anti-HBc positive. Viral DNA was confirmed in 4 employees, yielding a detection rate of 3.1% (95% CI 0.85–7.73%). This rate is consistent with global data, where OBI prevalence among HBsAg-negative HCWs ranges from 1 to 10% in endemic regions (34, 35). However, the concurrent immunological and epidemiological characteristics of these individuals are highly concerning. First, the median anti-HBs antibody concentration in OBI-positive individuals was critically low, at only 7 mIU/mL (IQR 0–15 mIU/mL), compared to 165 mIU/mL (IQR 55–480 mIU/mL) in their PCR-negative counterparts (*p* = 0.003). This places these OBI-positive individuals in the seronegative category, a phenomenon not uncommon among healthcare workers but representing a persistent epidemiological challenge (14, 36). Second, 75% of the OBI-positive staff were classified as having a low occupational risk according to official job classifications. The prevalence of anti-HCV antibodies in the study population was 5% (83 individuals), which is nearly double the rates reported in the general population (2.7–2.8% according to screening data of target cohorts in the Russian Federation for 2024–2025) and globally (7, 37). The current screening protocol for HCWs in Russia mandates HCV testing at least once every 12 months (15). Of the 83 anti-HCV-seropositive employees, HCV RNA was detected in only 3 (3.6%), corresponding to an overall prevalence of active HCV infection of 0.18% in the study population. This substantial discordance – where 96.4% of seropositive individuals lacked viral RNA in their blood – may be explained by several factors. It is well established that 15–45% of infected individuals undergo spontaneous clearance of acute hepatitis C while retaining lifetime anti-HCV antibodies (anti-HCV IgG) (19, 20). Furthermore, direct-acting antiviral agents, which exhibit high efficacy in treating various forms of chronic hepatitis C, have entered widespread clinical practice (19, 20), allowing complete viral elimination. It should be emphasized, however, that none of the study participants were aware of their status prior to this study or had received appropriate antiviral therapy. The poor predictive performance of our binomial logistic regression model for anti-HCV (AUC = 0.546; *p* = 0.68) underscores that HCV infection among HCWs lacks a clear association with age, sex, or official department profile. In contrast to hepatitis B, where age served as a strong predictor due to the vaccination implementation milestone, no vaccine exists for HCV. Consequently, the risk of infection is diffusely distributed and is likely driven by community-acquired exposure or accidental occupational injuries, which occur with similar probability across all department types.

The current mandatory screening of HCWs, which relies solely on HBsAg detection, is epidemiologically incomplete (15). It should be supplemented by mandatory anti-HBc antibody testing, as well as PCR diagnostics for vulnerable groups. Furthermore, because OBI and active HCV cases were identified among “low-risk” employees, standard precautions must be strictly adhered to by all healthcare facility personnel without exception.

### Strengths and limitations of the study

A key strength of this study is the large sample size, which encompasses employees across various occupational categories and clinical profiles. The application of multivariable statistical modeling allowed us to control for the confounding effect of age and identify true predictors of immune status. Another major advantage is the verification of occult viral hepatitis forms using highly sensitive real-time PCR assay systems. Although the study achieved the required sample size, the convenience-based nature of participation may introduce healthy worker bias. The predominance of female participants (92.5%) limits the ability to assess sex-specific associations. The finding that sex was not a statistically significant predictor of anti-HBc positivity (*p* = 0.299) should be interpreted with caution due to insufficient male representation rather than as definitive evidence of no effect. Information on the total years of healthcare employment was not systematically collected for all participants, which limits the ability to distinguish between age-related and duration-of-employment effects.

Among the limitations, the cross-sectional design of the study does not permit an assessment of antibody waning kinetics over time. Additionally, due to logistical and financial constraints, PCR diagnostics for OBI were restricted to a subsample of 129 individuals from a single facility, which warrants caution when extrapolating the precise OBI rate to the entire HCW population of the region. Among the limitations, we note that the sampling followed a census-based voluntary approach rather than probability-based random sampling, which may introduce participation bias. The sample size was calculated *a priori*; however, the convenience element of voluntary participation during screening sessions should be acknowledged. Finally, we lacked detailed historical records on vaccination regimens (vaccine types, intervals between doses) for older employees, which could have further refined our mathematical models.

## Conclusion

In this cross-sectional study, we found a high seroprevalence of HBV exposure markers, suboptimal anti-HBs seroprotection, and detection of HBV DNA in a small proportion of anti-HBc-positive individuals. These findings suggest gaps in current HBsAg-only screening practices. However, the cross-sectional design, single-facility setting for PCR testing, and small number of HBV DNA-positive cases limit causal inference. Larger prospective studies are needed to confirm these findings and evaluate the clinical significance of HBV DNA detection in this population. Based on our findings, we propose the following recommendations: (1) Transition from HBsAg-only screening to triple-marker testing (HBsAg, anti-HBc, anti-HBs) for HCWs, as recommended by CDC 2023 guidelines; (2) Implementation of post-vaccination anti-HBs monitoring 1–2 months after the primary vaccination series, with booster doses for non-responders; (3) Targeted PCR screening for HBV DNA in anti-HBc-positive HCWs, particularly those with low anti-HBs levels; (4) Extension of screening protocols to all facility staff, including those classified as low occupational risk; (5) Periodic reassessment of anti-HBs levels in vaccinated HCWs, especially in endemic settings.

## Data Availability

The original contributions presented in the study are included in the article/supplementary material, further inquiries can be directed to the corresponding author/s.
